# Pelvic aneurysmal bone cyst

**DOI:** 10.2349/biij.7.4.24

**Published:** 2011-10-01

**Authors:** MIA Sharifah, MH Nor Hazla, A Suraya, SP Tan

**Affiliations:** 1 Department of Radiology, Hospital Universiti Kebangsaan Malaysia, Kuala Lumpur, Malaysia; 2 Department of Orthopaedic, Hospital Universiti Kebangsaan Malaysia, Kuala Lumpur, Malaysia

**Keywords:** Aneurysmal bone cyst, Ilium, Pelvic bone

## Abstract

This paper describes an extremely rare case of a huge aneurysmal bone cyst (ABC) in the pelvis, occurring in the patient’s 5^th^ decade of life. The patient presented with a history of painless huge pelvic mass for 10 years. Plain radiograph and computed tomography showed huge expansile lytic lesion arising from the right iliac bone. A biopsy was performed and histology confirmed diagnosis of aneurysmal bone cyst. Unfortunately, the patient succumbed to profuse bleeding from the tumour.

## INTRODUCTION

Aneurysmal bone cyst (ABC) is a rare benign non-neoplastic expansile, vascular, locally destructive, osteolytic bone lesion [1, 5]. It is characterised by a reactive proliferation of connective tissue containing multiple blood-filled cavities. Presumably due to the local haemodynamic disturbances, the process arises *de novo* in bone or is engrafted on pre-existing bone lesions [6]. It exhibits a slight female preponderance. The most common sites are the metaphysio-epiphyseal area of long bones or vertebrae with eccentric expansion [1]. Vast majority (about 80%) of patients with ABC are below 20 years of age. Pelvic or iliac ABC has rarely been reported to occur in patients with relative old age [1]. This paper describes a rare case of a huge ABC in the iliac bone occurring in the 5^th^ decade of life.

## CASE REPORT

A 51-year-old female presented with a ten-year history of right inguinal and pelvic mass. This painless mass gradually increased in size. She had been under orthopaedic outpatient follow-up ten years ago for the right pelvic mass. However, she defaulted follow-up and went for traditional medicine. In the past two years, the mass rapidly grew larger, limiting her movement and physical activity. There was no weakness of the right lower limb and there was no significant history of loss of weight or appetite. Physical examination revealed a palpable hard and fixed mass in the right inguinal, upper hip and right flank region over the anterior superior iliac spine (ASIS). The right femoral and distal pulses were normal. There was no local heat and erythema. The movement of the right hip was restricted by this huge mass. Power and sensation of the lower limb remained normal.

**Figure 1:**
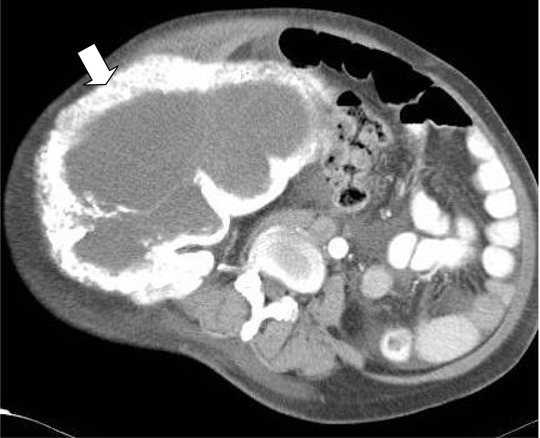
Contrast-enhanced computed tomography of the pelvis and the abdomen showed huge expansile lytic lesion (arrow) arising from the illium and ischium. The bowels are displaced to the left. Small area of soft tissue component (open arrow). The cortex is thickened and irregular. The lower abdomen and the pelvis were tilted by this huge mass.

Plain radiograph of the pelvis showed a huge expansile lytic lesion of the right iliac wing. Contrasted computed tomography of the abdomen and pelvis demonstrated a huge expansile lytic lesion arising from the right ilium and ischium. It measured 25 cm (width) × 25 cm (height) × 23 cm (craniocaudal). The lower abdomen and the pelvis were tilted by this huge mass (Figure 1). The Hounsfield unit of the mass was 15, which was consistent with cystic mass. There was no fluid-fluid level seen within. The bony cortex of the pelvic bone was irregularly thickened with areas of endosteal scalloping. There was no chondroid matrix seen within. The bowels were displaced to the left; however, there was clear demarcation between this mass and the bowel. The right femoral artery and the right femoral vein were intact. There was also minimal ascites.

Patient had major difficulty to walk because of the increasing size of the tumour. Based on a corporate social responsibility programme patient was transferred to a private medical centre for further diagnostic and therapeutic management.

Preoperatively a contrasted MRI was performed. Several differential diagnoses were discussed, such as primary ABC, ABC secondary to giant cell tumour or primary malignant tumours like osteosarcoma and chondrosarcoma.

An open biopsy was planned and performed to obtain representative tissue for further histopathological investigation. The diagnosis of aneurysmatic bone cyst was established by frozen section (Figure 3).

**Figure 2:**
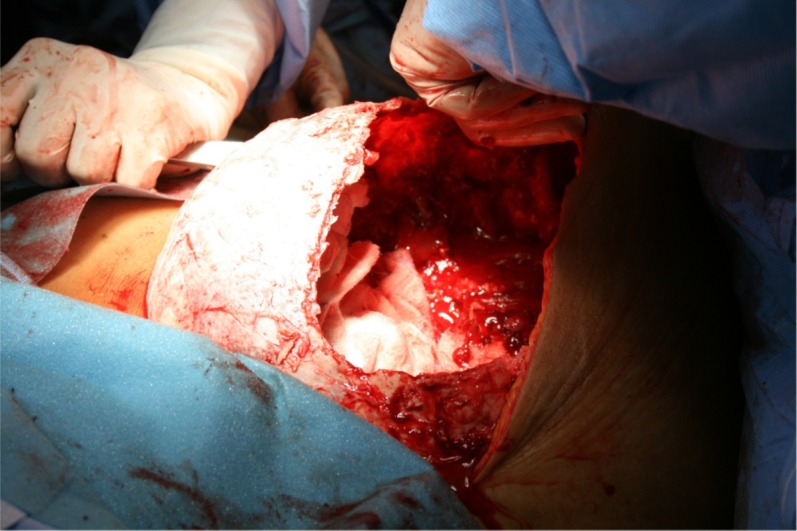
Intraoperative photograph shows the "blowout" iliac bone after removal of the ventrolateral wall.

Intraoperative, there was a large cavity in the pelvic bone, filled with hemorrhagic liquid. The liquid within the lesion had a tamponade effect, such once the cavity was opened, there was a severe diffuse bleeding. The wall of the cavity was like a coconut shell (Figure 2). To reduce and control the bleeding surface the ventral part of the lesion was removed, which did not improve the bleeding situation. An amputation was definitely refused by patient preoperatively. Unfortunately the patient succumbed to perfuse bleeding despite extensive blood transfusion and resuscitation after transfer to the intensive care unit. 

**Figure 3:**
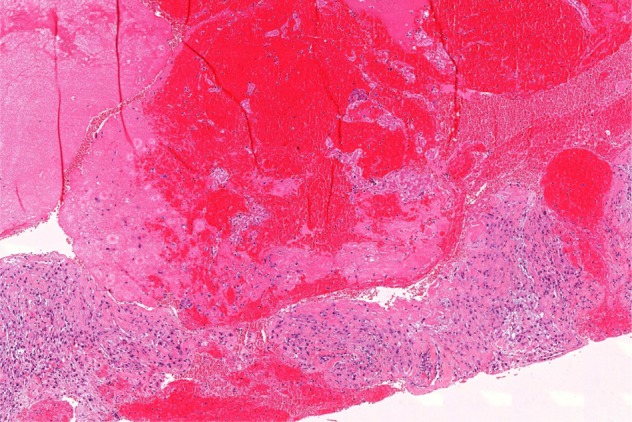
Histological features composed of haemorrhagic spaces of blood-filled cavities surrounded by fibrotic septa, inflammatory cells and osteoclast cells that are distributed around the cystic spaces (HE). x5 x10

## DISCUSSION

Aneurysmal bone cyst is a non-neoplastic expansile bone lesion consisting of blood-filled spaces separated by connective tissue septa containing bone or osteoid and osteoclast giant cells [2]. Ninety percent of patients with ABC present before the age of 30 [1]. This case manifested several unique characteristics which include; occurrence in older age group and unusual size of the lesion, which measured 25 × 25 × 23 cm. This is the largest ABC of iliac ever reported. The previous reported case was in 2002, occurring in a 42 year-old female with a lesion the size of 10.0 × 9.0 × 8.5 cm in the iliac bone [1].

A study of pelvic bone cysts by Hammound *et al*. revealed that the majority of bone cysts occur in the ilium, followed by pubic ramus and ischium, respectively. They also found that the posterior part of the illium, which is adjacent to the sacral iliac joint, occurs predominantly in the older age group [4].

Radiologically, ABCs from pelvis usually demonstrate a fusiform expansile “blowout” lesion. Sometimes, an expansile, lobulated, lytic, multiseptated cystic lesion will contain fluid-fluid level. Our patient demonstrated expansile, “blowout”, lytic changes; however, there was no fluid- fluid level which is the characteristic feature of ABC. Although the findings mentioned above may suggest ABC, it also may suggest many other disease entities such as osteosarcoma and giant cell tumour [2, 5].

ABCs, especially when they present in an aggressive manner, can sometimes be difficult to distinguish from a telangiectatic osteosarcoma. Although telangiectatic osteosarcoma mimics this gross morphologic feature, it is more common in patients over 20 years of age [1].

These diagnostic problems are due to the rapid growth of ABC and its extensive destruction of bone. The diagnosis becomes even more complicated if there is an extra-osseous and soft-tissue tumour mass [2]. Preoperative MRI may also be helpful and may demonstrate the fluid-fluid level, which is characteristic of ABC features in MRI. MRI is also very useful for preoperative planning.

The main treatment option is surgery. Enneking classified the surgical interventions into three types: (1) intralesional (curettage and bone grafting), (2) marginal (en bloc) resection, and (3) wide resection (segmental resection). In preoperative planning, the location and growth pattern of the ABC are of utmost importance. It is most advantageous if the ABC grows superficially and involves no more than one-third of the bone width. These cases are suitable for intra-lesional excision to remove the cyst in normal bony tissue. This is a good intervention for ABC and results in normal joint function and few local recurrences. However, in sites such as the pelvis or spine or when the size of the cyst is particularly large, surgical treatment of extraperiosteal excision and bone grafting become difficult and risky. Thus, careful curettage and bone grafting still remain the surgical method of choice in such cases [1].

Other treatment options include curettage and bone graft, and low-dose radiotherapy [3]. Another option is preoperative embolisation, which is frequently used because ABC lesions are highly vascular.

Recurrence occurs most commonly during the first two post-operative years. The factors influencing recurrence are still unknown. Age, lesion location, lesion size, and number of mitotic figures have been suggested. Campanacci *et al.* have reported that 26% of patients with aggressive ABCs treated with curettage had a recurrence, whereas no patients treated with partial or complete resection had a recurrence [1].
